# Variations in Sexual Size Dimorphism in Two Anurans Along an Urbanization Gradient in Shanghai: Assessment of Rensch's Rule

**DOI:** 10.1002/ece3.73825

**Published:** 2026-06-15

**Authors:** Ben Li, Ningning Liu, Shurong Zhong, Tianhou Wang, Rongquan Zheng, Qing Song, Zhenghuan Wang, Wei Zhang

**Affiliations:** ^1^ College of Life Sciences, Zhejiang Normal University Jinhua China; ^2^ School of Life Science, East China Normal University Shanghai China; ^3^ Ministry of Education Key Laboratory for Biodiversity Science and Ecological Engineering, Coastal Ecosystems Research Station of the Yangtze River Estuary, School of Life Sciences Fudan University Shanghai China; ^4^ Shanghai Academy of Landscape Architecture Science and Planning Shanghai China; ^5^ Natural History Research Centre of Shanghai Natural History Museum Shanghai Science and Technology Museum Shanghai China

## Abstract

Variations in sexual size dimorphism (SSD) have important consequences for animal ecology, behavior, population dynamics, and the evolution of life‐history traits, and can be explained by many intraspecific and interpopulation hypotheses. Urbanization is an important factor that affects anuran phenotypic characteristics (including morphology), but how it impacts anuran SSD has been explored less widely. In this study, we aimed to investigate the differences in SSD in two dominant amphibian species populations; that is, the Hong Kong rice‐paddy frog ( *Fejervarya multistriata* ) and Beijing gold‐striped pond frog ( *Pelophylax plancyi* ), across an urban–rural gradient in Shanghai (including urban, suburban, and rural areas). We also tested whether these variations in SSD supported Rensch's rule or not. Our results showed that in both sexes, the snout‐vent length (SVL) in *F. multistriata* was higher in urban areas compared with suburban and rural areas. In addition, only male *P. plancyi* exhibited a higher SVL in urban areas compared with those in rural areas. Furthermore, we found that the SSD in SVL was significantly higher for urban populations than suburban and rural populations of *F. multistriata* . All SSD results in terms of SVL, head width, forelimb length, and hindlimb length in these two frog species supported the inverse Rensch's rule based on reduced major axis regression. Moreover, the size scaling rates in these two frog species were higher in urban and suburban populations than rural populations. Thus, we concluded that urbanization shapes SSD in these two anurans according to the inverse of Rensch's rule, and that variations in morphological characteristics, SSD, and size scaling rates differed between these two anuran species regarded as an “urban‐associated species” ( *P. plancyi* ) and “urban‐sensitive species” ( *F. multistriata* ).

## Abstract 2

In Shanghai, sexual size dimorphism in snout–vent length for urban populations of *Fejervarya multistriata* was significantly higher than that in the suburban and rural populations. In contrast, no significant difference was observed among populations of *Pelophylax plancyi* along the urban‐rural gradient. All sexual size dimorphism of snout–vent length, head width, forelimb length, and hindlimb length in two frog species support the inverse of Rensch's rule.

## 1 Introduction

Sexual dimorphism in animals refers to the phenomenon where different sexes in the same species differ in terms of body size, body color, local morphological features, and even physiology, behavior, and life history, which is an important aspect of animal evolution (Darwin 1871; Shine 1979). In particular, sexual size dimorphism (SSD) in terms of body size is widely present in animals and has received great attention from researchers. Traditional sexual selection theory predicts that SSD is male‐biased, driving larger male body size when mating success by males is positively correlated with their body size during contests with other males and female choice (Darwin 1871; Andersson 1994). Alternatively, fecundity selection theory predicts that SSD is female‐biased in species where fitness increases with larger brood size (transient fecundity) attained by females of greater size (Shine 1988). However, this prediction has been questioned because it ignores the prevalent and simultaneous effects of sexual selection for larger (or smaller) male size (Pincheira‐Donoso and Hunt 2017). Therefore, larger females that cause female‐biased SSD can evolve due to mechanisms other than fecundity selection, and female‐biased SSD is not necessarily correlated with the strength of fecundity selection (Cox et al. 2003; Pincheira‐Donoso et al. 2021). Moreover, niche divergence theory predicts that intersexual competition for ecological resources creates natural selection that drives divergence between males and females, which has increasingly gained support (Shine 1989; Bolnick and Doebeli 2003; Pincheira‐Donoso et al. 2018). According to this hypothesis, the intensity of intraspecific ecological competition (including for water, food, and habitat selection) is mitigated by disruptive natural selection driving males and females into different regions of the niche space (Temeles et al. 2000; Bolnick and Doebeli 2003; Meiri et al. 2014).

In general, SSD plays a significant role in understanding anuran life history evolution and mating systems (Shine 1979; Martins et al. 2018). Previous studies of sexual dimorphism in anurans mostly focused on understanding the intensity of sexual dimorphism in the morphology of a single or several anuran species (Liao et al. 2015; Reinhard et al. 2015; Dursun and Özdemir 2022), and some also focused on changes in the motor ability and body color of anurans (Rojas and Endler 2013). Moreover, a large number of anuran species exhibit variations in SSD over geographical (Pearson et al. 2002; McGarrity and Johnson 2009; Yu et al. 2010), altitudinal (Feng et al. 2015; Dursun et al. 2022; Gül et al. 2024), and climatic gradients (Goldberg et al. 2018; Dursun et al. 2023). Furthermore, differences in microhabitat conditions (Stuart‐Fox and Moussalli 2007), nesting sites (Pincheira‐Donoso et al. 2021), fecundity (Shine 1979), parental care behavior (Han and Fu 2013), life history and age‐related parameters (Monnet and Cherry 2002; Seglie et al. 2010; Üzüm et al. 2021), and human activity (Lambert et al. 2015) are regarded as important factors that can affect SSD in anurans. Therefore, some complex selective pressures may shape SSD in anurans globally, and they remain largely unexplored, especially in some female‐biased anuran species (Kupfer 2007).

Rensch's rule describes an allometric relationship between SSD and body size among species, when considering that body size divergence is greater among males than females, and thus the degree of SSD increases with body size in species with male‐biased SSD and decreases with body size in species with female‐biased SSD (Rensch 1950; Abouheif and Fairbairn 1997). Some supporting evidence has been reported in female‐biased SSD species (Liao et al. 2013; Webb and Freckleton 2007), but exceptions exist showing the inverse of Rensch's rule, that is, the degree of SSD increases with female body size among female‐biased SSD ectotherms (Herczeg et al. 2010; Liao et al. 2015). For example, Liao et al. (2013) indicated that 36 species with female‐biased SSD, and three species with male‐biased SSD failed to support Rensch's rule, including when the analyses were phylogenetically corrected. In fact, Rensch's rule is comprehensively affected by sexual selection, natural selection, and developmental constraints in amphibians (Liao et al. 2013; Colleoni et al. 2014; Reyes‐Puig et al. 2023), and fecundity selection on females favoring large size balances out sexual selection in favor of large male size, thereby failing to support Rensch's rule (Liao et al. 2013). The rule was originally formulated as a macroevolutionary and interspecific pattern, and thus the application of Rensch's rule has generally been appropriately conducted at the interspecific level (Colleoni et al. 2014; Cox et al. 2003; Székely et al. 2004). It should also be noted that intraspecific level studies were also conducted along environmental gradients (Blanckenhorn et al. 2006; Fairbairn 2005; Lengkeek et al. 2008; Liao 2013), such as altitudinal and latitude gradients. Critically, divergent selection pressures act on different morphological traits, leading to variations in SSD in terms of both its direction and intensity relative to body size (Ivanović et al. 2025). In particular, some researchers have shown that allometry parameters significantly reflect SSD in anurans (Sanger et al. 2013; Kaliontzopoulou et al. 2015; Adams et al. 2020).

Globally, urbanization continues to increase, dramatically and irreversibly transforming landforms through habitat loss and fragmentation, together with a rise in impervious surfaces (United Nations 2022; Yang and Wu 2022) and further affecting urban biodiversity (Rainey et al. 2024). These alterations to both biotic (e.g., biological community structure) and abiotic factors (e.g., landscape and microhabitat elements) can significantly affect the phenotypic characteristics of amphibians, including their morphology, physiology, and behavior (Thompson et al. 2022), as well as genetic traits (Wei et al. 2021; Fusco et al. 2021). Given their unique human‐altered features, urban environments are considered to increase or favor mechanisms that increase sexual differences in animals by altering the resource extraction process and allocation to the sex with more variable phenotypes (Chulisov et al. 2019; Huang et al. 2025). Liu et al. (2025) indicated that *Fejervarya multistriata* exhibited significant variations in life span, age structure, and snout–vent length (SVL) along a urban–rural gradient in Shanghai, where urban and suburban individuals were younger with a larger SVL compared with those in rural areas. In fact, some studies have shown that SSD in anurans can be explained by age differences and developmental time differences between males and females (Monnet and Cherry 2002; Liao et al. 2013). Thus, we assume that these phenotypic and genetic changes in anurans, as well as biotic and abiotic elements, will further affect SSD variations. However, few studies have focused on the changes in SSD in amphibians under urban environments, which are important factors that affect population ecology and biology in anurans. For example, Ofori et al. (2025) found that the African common toad ( *Sclerophrys regularis* ) exhibited female‐biased sexual dimorphism in terms of the snout‐vent length (SVL), body mass, and head volume, where the extent of SSD was strongest for a suburban population in the Accra Plains region of Ghana, and the selective pressures that acted differently on male and female individuals to maximize their fitness were stronger in the suburban area than urban and rural areas (Thompson et al. 2022). To the best of our knowledge, few studies have investigated the relationships between sexual dimorphism, allometry, and Rensch's rule with urbanization in anurans.

Thus, we selected Shanghai city as a typical representative area that has undergone rapid urbanization in China, with the anuran population size also decreasing recently (Zhang et al. 2016). The physiological and behavioral characteristics of anurans have also changed along the urbanization gradient (Li et al. 2016; Liu et al. 2022). Two dominant amphibian species in Shanghai were chosen as the study species: *Fejervarya multistriata* and *Pelophylax plancyi* . Previous research has shown that their morphological characteristics vary due to urbanization (Zhong et al. 2023; Liu et al. 2025), and Liao et al. (2013) obtained evidence for *F. multistriata* that was consistent with the inverse of Rensch's rule.

The aims of this study were to: (1) compare the differences in SSD for SVL and other morphological characteristics (i.e., head width, forelimb length, and hindlimb length) along the urbanization gradient in Shanghai; and (2) further test trends in terms of Rensch's rule and the size scaling rates of morphological characteristics along the urbanization gradient. The findings obtained in this study help us to further understand the effects of urbanization on the population dynamics, reproductive strategies, and evolutionary strategies of amphibians.

We hypothesized that SSD would change along the urban–rural gradient due to differences in environmental pressure caused by urbanization (Thompson et al. 2022), and according to the inverse Rensch's rule (Liao 2013; Liao et al. 2013), that SSD would decrease with increasing urbanization due to the greater body size of male individuals being affected by resource and reproductive pressures (Zhong et al. 2023), and lower life span of female individuals (Liu et al. 2025) in the urban environment than suburban and rural environments. Moreover, the variations in SSD (including morphological characteristics and size scaling rates) due to the effects of urbanization could be smaller in *P. plancyi* than *F. multistriata* because the former is an “urban‐associated” species whereas the latter is an “urban‐sensitive” species (Hamer and McDonnell 2008).

## 2 Materials and Methods

### 2.1 Study Area and Urbanization Gradient

Shanghai (30°40″ N to 31°53″ N and 120°51″ E to 122°12″ E) is located in the southeastern Yangtze River Delta in eastern China, and covers a total area of 6340.5 km^2^ with a population of 24.3 million. Shanghai is one of the largest cities in the world and continues to undergo rapid urbanization. Given its vast size, Shanghai has many rivers, lakes, and paddy rice fields that are suitable habitats for amphibians (Liu et al. 2022, 2025).

**FIGURE 1:**
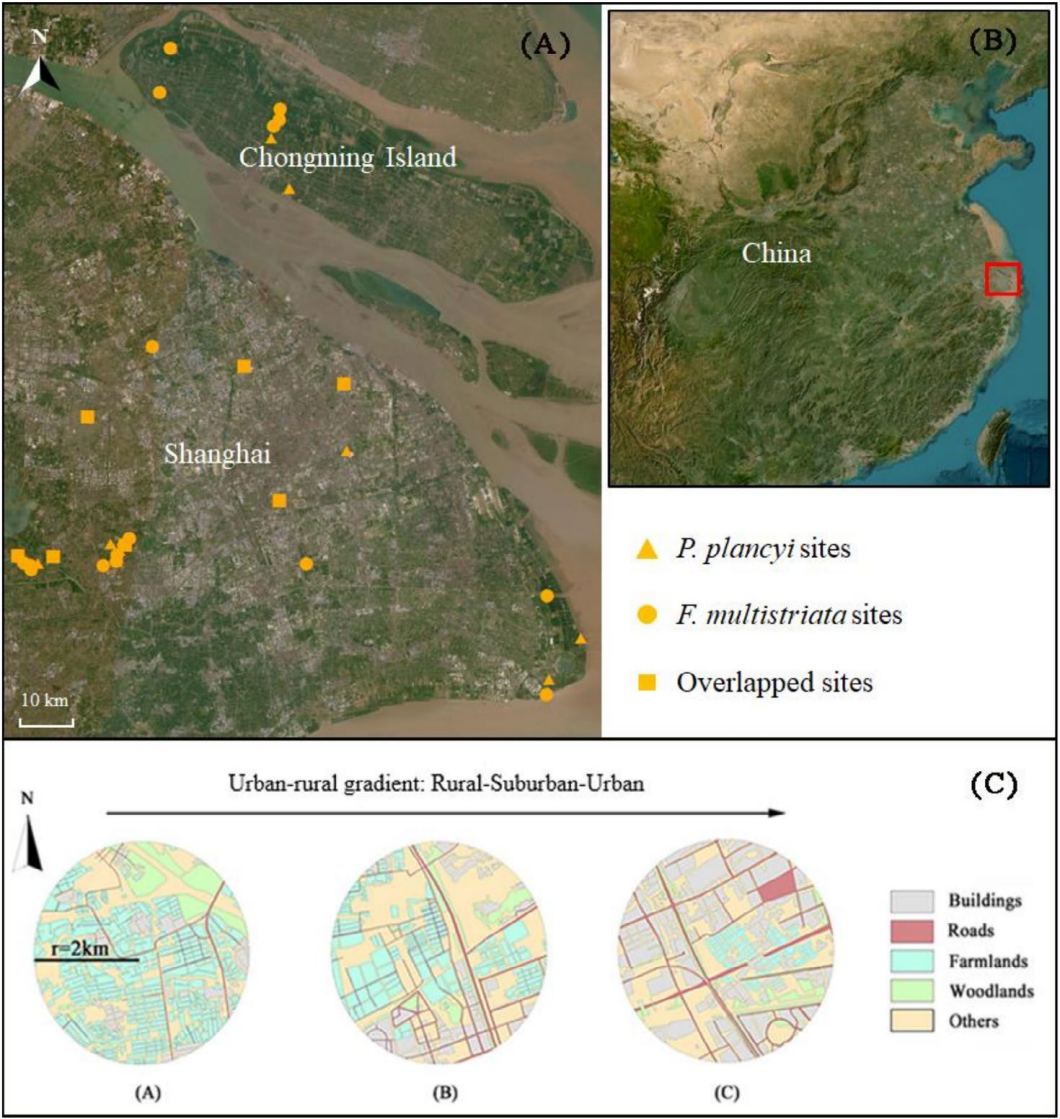
Locations of sampling sites for *Fejervarya multistriata* and *Pelophylax plancyi* along the urban–rural gradient in Shanghai.

In total, 287 *F. multistriata* (SVL > 25 mm) and 154 *P. plancyi* (SVL > 35 mm) adults (Figure S1) were randomly captured across 34 study sites (24 sites for *F. multistriata* and 17 sites for *P. plancyi* ) along an urban–rural gradient in Shanghai during their breeding season from April to August in 2020 and 2021 (Figure 1). The frogs were anesthetized with MS222, and these individuals were released after obtaining measurements. Land‐use data for these study sites were acquired from Google Earth satellite images obtained in September 2020 with ArcGIS 10.3 (ESRI, Redlands, CA, USA). Within a 2‐km radius of each sampling site, four land use/cover types were defined with Fragstats 4.2 (McGarigal et al. 2012) as: farmlands, woodlands, buildings, and aquatic areas. The distance between the edges of each adjacent study site (the edge of each landscape) was > 1 km to ensure spatial independence (Li et al. 2020, 2024). The urban index was used to define the urbanization gradient for each study site based on principal component analysis with these four land‐use types, where the first principal component was used to define the urban index for each study site (Zhang et al. 2016; Liu et al. 2025). To increase the linearity of the data, the urban index for each study site was transformed to a scale of 0–1.

All study sites along the urbanization gradients were classified as rural (< 0.30), suburban (0.3–0.70), or urban (> 0.70) based on their urbanization score (Figure 1). The sample sizes at urban, suburban, and rural sites for *F. multistriata* were three, nine, and 12, respectively. The sample sizes at urban, suburban, and rural sites for *P. plancyi* were five, five, and seven, respectively. The minimum sample size for each study site was three per sex, although samples were scarce at some urban study sites.

### 2.2 Sex Determination and Morphological Measurement

The sex of individual anurans was determined based on the presence or absence of nuptial pads on their toes (Li et al. 2020). The SVL, head width, forelimb length, and hindlimb length were also measured for individual anurans because they are functionally relevant, where the limb lengths are related to locomotion and amplexus, head width to feeding, and SVL to overall body size, competitive force, and fecundity (Zhong et al. 2023). Measurements were taken with an electronic digital caliper (0.1 mm) (Li et al. 2016, 2019, 2020). To eliminate potential correlations between other morphological traits (head width, forelimb length, and hindlimb length) and SVL (Liu et al. 2025), residual analysis was used to transform the other morphological traits, and the data were normalized to a scale of 0–1.

### 2.3 SSD

SSD was estimated using the sexual dimorphism index [SDI = (size of female population/size of male population) − 1], which was defined as positive when females were the larger sex and negative when males were the larger sex (Lovich and Gibbons 1992). Larger SDI values indicated greater differences between anuran sexes and vice versa. We also investigated changes in the intensity of sexual dimorphism for other morphological traits (SVL, head width, forelimb length, and hindlimb length) along the urbanization gradient.

### 2.4 Data Analysis

Kolmogorov–Smirnov and Levene's tests were conducted to assess the normality and homogeneity of variances for all data, respectively. To consider the potential correlations between SVL and other morphological traits, Pearson's correlation coefficients were calculated if the measured data satisfied the assumptions of normality and homogeneity of variances; otherwise, Spearman's correlation coefficients were calculated. To avoid any potential autocorrelations between SVL and other morphological traits, linear regression equations were used with SVL as the dependent variable and other morphological traits as independent variables.

For normally distributed data, one‐way analysis of variance was conducted followed by Tukey's HSD tests to assess differences in the morphological traits consisting of SVL, head width, forelimb length, and hindlimb length among different urbanization gradients for *P. plancyi* and *F. multistriata* individuals, as well as the related intensity of sexual dimorphism in populations among different urbanization gradients. For non‐normally distributed data, the Kruskal‐Wallis test was conducted following by pairwise Mann–Whitney *U* tests. The differences between the four morphological traits in female and male sexes along the urban–rural gradients were assessed by using *t*‐tests and Mann–Whitney *U* tests. Moreover, we conducted reduced major axis (RMA) regression of log_10_ (male size) against log_10_ (female size) using the population means of the morphological traits to test Rensch's rule, where the head width, forelimb length, and hindlimb length were log_10_ (~+1) transformed. When log_10_ (female size) was plotted on the x‐axis and log_10_ (male size) on the y‐axis, a slope larger than 1.0 provided evidence for Rensch's rule (Fairbairn 1997).

Linear regression was performed using SVL and the other morphological traits for the two anuran sexes along the urbanization gradient. The slopes of these regressions were used to reflect the intersexual size scaling rates of males and females in different urbanized areas. Subsequently, the differences in the sexual size scaling rates for each and all morphological traits were compared across the urbanization gradient by calculating the differences in the slopes between females and males (female slope minus male slope). RMA regression was conducted by using the “*smart*” package in R (version 3.4‐8, Warton et al. 2012). All statistical analyses were performed using R 4.2.3 (R Development Core Team 2022).

## 3 Results

### 3.1 Morphology and SSD Along the Urbanization Gradient

The SVL, head width, forelimb length, and hindlimb length measurements in urban, suburban, and rural areas for all *F. multistriata* and *P. plancyi* individuals are shown in Table 1. The SVL measurements for male and female *F. multistriata* and *P. plancyi* correlated significantly with their head width, forelimb length, and hindlimb length according to Pearson's correlation coefficients (Table S1). Therefore, the formulae used to determine head width, forelimb length, and hindlimb length in *F. multistriata* and *P. plancyi* based on the residual method are shown in Table S2. Among the morphological characteristics, we found that both male and female urban *F. multistriata* individuals had longer SVL measurements than suburban (male, *p* < 0.001; female, *p* < 0.001) and rural (male, *p* < 0.001; female, *p* < 0.001) individuals, rural *F. multistriata* individuals had longer SVL measurements than suburban individuals (male, *p* = 0.003; female, *p* = 0.003), and male *P. plancyi* individuals had longer SVL measurements in urban areas than rural areas (*p* = 0.041) (Table 1).

**TABLE 1:** Snout‐vent length, head width, forelimb length, and hindlimb length measurements in urban, suburban, and rural areas for all *F. multistriata* and *P. plancyi* individuals (mean and standard deviation).

Sex	Urban					Sub‐urban				Rural		
	Snout–vent length	Head width	Forelimb length	Hindlimb length	Snout–vent length	Head width	Forelimb length	Hindlimb length	Snout–vent length	Head width	Forelimb length	Hindlimb length
***F. multistriata***												
Male (*N* = 133)	**41.191** ^**a**^ (1.827)	14.379^a^ (1.904)	16.389^a^ (1.904)	58.619^a^ (7.529)	**35.101** ^**c**^ (3.180)	12.139^a^ (1.248)	13.767^a^ (1.353)	49.521^a^ (4.889)	**36.875** ^**b**^ (2.808)	12.109^a^ (1.269)	13.745^a^ (1.376)	49.442^a^ (4.971)
Female (*N* = 154)	**47.913** ^**a**^ (8.344)	13.930^a^ (1.787)	15.393^a^ (1.495)	57.109^a^ (6.468)	**35.854** ^**c**^ (4.728)	11.587^a^ (1.054)	13.180^a^ (1.142)	47.401^a^ (4.127)	**38.724** ^**b**^ (5.094)	12.842^a^ (1.112)	14.54^a^ (1.205)	52.314^a^ (4.353)
***P. plancyi***												
Male (*N* = 61)	**42.446** ^**a**^ (3.30)	17.548^a^ (3.029)	21.989^a^ (4.184)	75.131^a^ (11.940)	42.063^ab^ (6.958)	14.559^a^ (1.827)	18.197^a^ (3.077)	60.214^a^ (8.788)	**38.645** ^**b**^ (4.085)	14.971^a^ (3.238)	18.628^a^ (4.251)	61.456^a^ (15.416)
Female (*N* = 93)	50.041^a^ (10.065)	14.182^a^ (1.889)	18.198^a^ (2.458)	60.258^a^ (8.719)	46.593^a^ (12.176)	15.857^a^ (3.904)	20.361^a^ (4.305)	66.000^a^ (14.464)	43.458^a^ (12.144)	14.869^a^ (3.761)	19.145^a^ (4.028)	61.623^a^ (12.256)

*Note:* Different lowercase letters indicate significant differences between areas at *p* < 0.05 based on Tukey's HSD test. Bold text indicates significant differences.

Most of the SDIs for different morphological traits in the two anuran species were positive in urban, suburban, and rural areas, indicating female‐biased sexual dimorphism for these morphological traits (Tables 2 and 3). However, the SDIs were negative for forelimb length in the urban environments for *F. multistriata* (Table 2), head width in the suburban environment, and hindlimb length in rural and suburban environments for *P. plancyi* , indicating male‐biased sexual dimorphism for these morphological traits (Table 3).

**TABLE 2:** Sexual size dimorphism index (SDI) and *t*‐test or Mann–Whitney *U* tests results to compare snout–vent length, head width, forelimb length, and hindlimb length measurements between all female and male *F. multistriata* individuals in urban, suburban, and rural areas.

Morphological characteristics	Urban (*N* = 24)		Suburban (*N* = 128)		Rural (*N* = 135)	
	SDI	*p*	SDI	*p*	SDI	*p*
Snout‐vent length	0.163	***z* = −3.071, *p* = 0.001**	0.021	*z* = −0.436, *p* = 0.663	0.028	***z* = −3.297, *p* = 0.001**
Head width	0.043	*t* = −0.295, *p* = 0.771	0.024	*t* = −0.810, *p* = 0.419	0.019	*t* = 0.791, *p* = 0.430
Forelimb length	−0.052	*t* = 0.425, *p* = 0.675	0.086	*t* = −1.774, *p* = 0.079	0.111	***t* = 3.218, *p* = 0.002**
Hindlimb length	0.235	*t* = −1.036, *p* = 0.311	0.411	***t* = −4.164, *p* < 0.001**	0.264	***t* = 4.571, *p =* 0.001**

*Note:* Bold text indicates significant differences.

**TABLE 3:** Sexual size dimorphism index (SDI) and *t*‐test or Mann–Whitney *U* test results to compare snout–vent length, head width, forelimb length, and hindlimb length measurements between all female and male *P. plancyi* individuals in urban, suburban, and rural areas.

Morphological characteristics	Urban (*N* = 36)		Suburban (*N* = 48)		Rural (*N* = 70)	
	SDI	*p*	SDI	*p*	SDI	*p*
Snout‐vent length	0.179	***z* = −2.816, *p* = 0.004**	0.104	*t* = 1.440, *p* = 0.157	0.124	*z* = −1.244, *p* = 0.213
Head width	0.089	*t* = 0.652, *p* = 0.519	−0.106	*t* = −1.052, *p* = 0.298	0.087	*t* = 0.541, *p* = 0.591
Forelimb length	0.331	***z* = −2.215, *p* = 0.027**	0.284	*t* = 1.604, *p* = 0.116	0.181	*z* = −0.386, *p* = 0.699
Hindlimb length	0.014	*t* = 0.330, *p* = 0.743	−0.033	*t* = −0.573, *p* = 0.570	−0.041	*t* = −1.275, *p* = 0.207

*Note:* Bold text indicates significant differences.

In addition, we found that the SVL (*p* = 0.001), forelimb length (*p* = 0.002), and hindlimb length (*p* = 0.001) measurements were significantly higher for female *F. multistriata* individuals than male individuals in rural environments (Table 2). The hindlimb length (*p* < 0.001) measurements were significantly higher for female *F. multistriata* individuals than male individuals in suburban environments, and the SVL measurements (*p* = 0.001) were significantly higher for female *F. multistriata* individuals than male individuals in urban environments (Table 2). Moreover, we found that the SVL (*p* = 0.004) and forelimb length (*p* = 0.027) measurements were significantly higher for female *P. plancyi* individuals than male individuals in urban environments (Table 3).

### 3.2 Variations in SSD Among Different Populations Along the Urbanization Gradient

**FIGURE 2:**
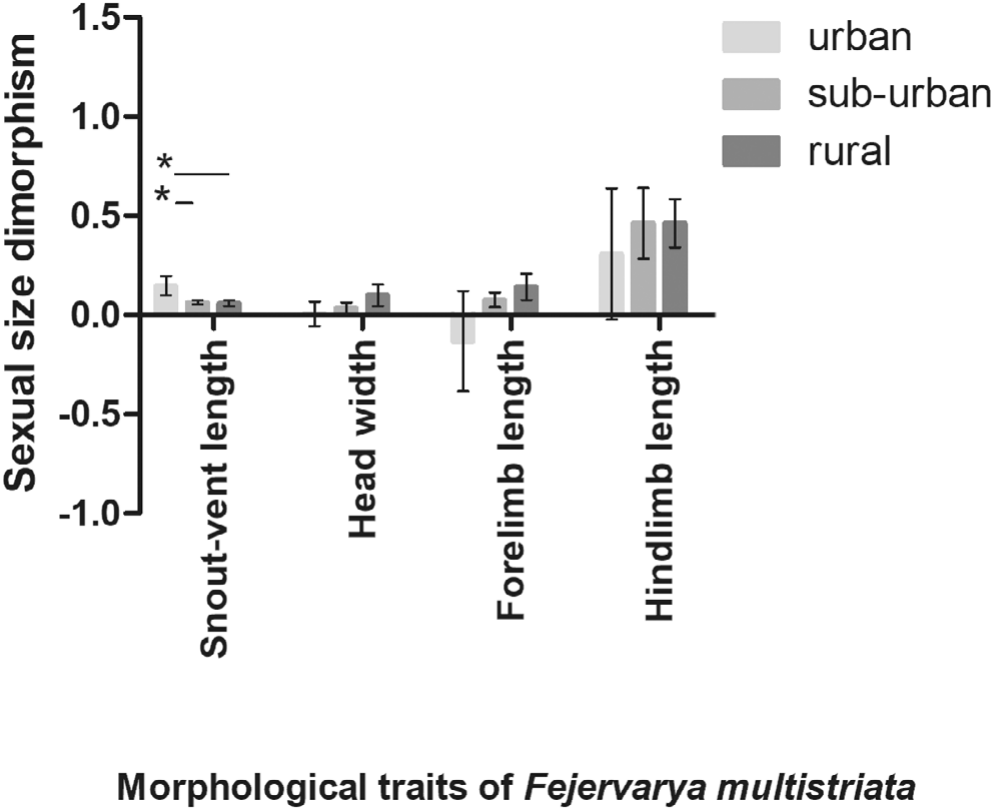
Comparison of the intensity of sexual dimorphism in *F. multistriata* in terms of snout‐vent length, head width, forelimb length, and hindlimb length measurements among urban (*N* = 3), suburban (*N* = 9), and rural (*N* = 12) populations (mean and standard deviation) (*p < 0.05).

**FIGURE 3:**
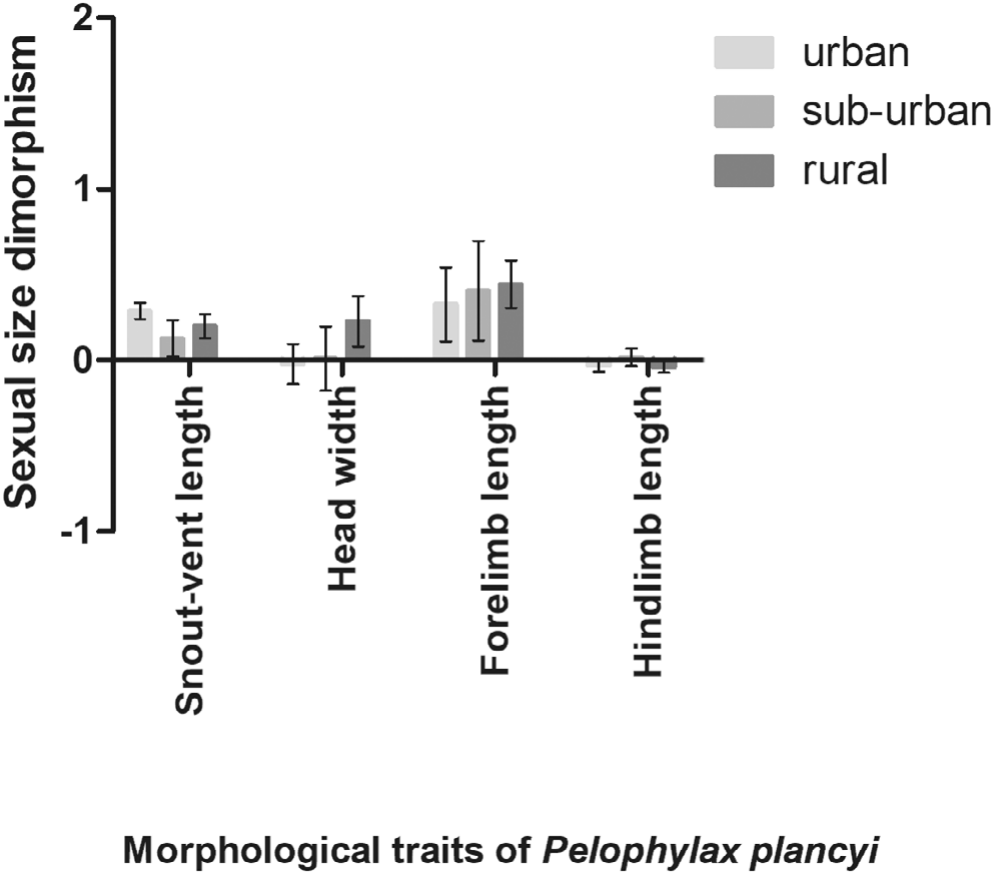
Comparison of the intensity of sexual dimorphism in *P. plancyi* in terms of snout‐vent length, head width, forelimb length, and hindlimb length measurements among urban (*N* = 5), suburban (*N* = 5), and rural (*N* = 7) populations (mean and standard deviation).

The intensity of SDI for SVL measurements was significantly higher in urban populations of *F. multistriata* than suburban (*p* = 0.022) and rural (*p* = 0.013) populations, but there was no significant difference in the intensity of SDI for each of the other morphological characteristics along the urbanization gradient in Shanghai (Figure 2 and Table S3). By contrast, there was no significant difference in the intensity of SDI for each morphological characteristic in *P. plancyi* populations in urban, suburban, and rural areas (Figure 3 and Table S3).

All SSDs for morphological characteristics in these two frog species did not support Rensch's rule (all slopes < 1), and they could defined as the inverse of Rensch's rule (Table 4).

**TABLE 4:** Reduced major axis regression of log_10_ (male size) against log_10_ (female size) across populations along the urban–rural gradient in Shanghai. Head width, forelimb length, and hindlimb length measurements were log_10_(~+1) transformed.

Morphological characteristics	Slope	*R* ^2^	95% CI	*p*
***F. multistriata*** **(*N =* 24)**				
Snout–vent length	0.578	0.602	0.390, 0.904	< 0.001
Head width	0.971	0.148	−0.108, 0.077	0.060
Forelimb length	0.661	0.172	0.002, 0.077	0.040
Hindlimb length	0.920	0.165	−0.082, 0.031	0.041
** *P. plancyi* (*N =* 17)**				
Snout–vent length	0.515	0.164	0.296, 1.169	0.107
Head width	0.703	0.306	−0.012, 0.054	0.021
Forelimb length	0.593	0.247	−0.025, 0.054	0.043
Hindlimb length	0.835	0.002	−0.062, 0.152	0.852

### 3.3 Comparison of Size Scaling Rates for Other Morphological Traits

**FIGURE 4:**
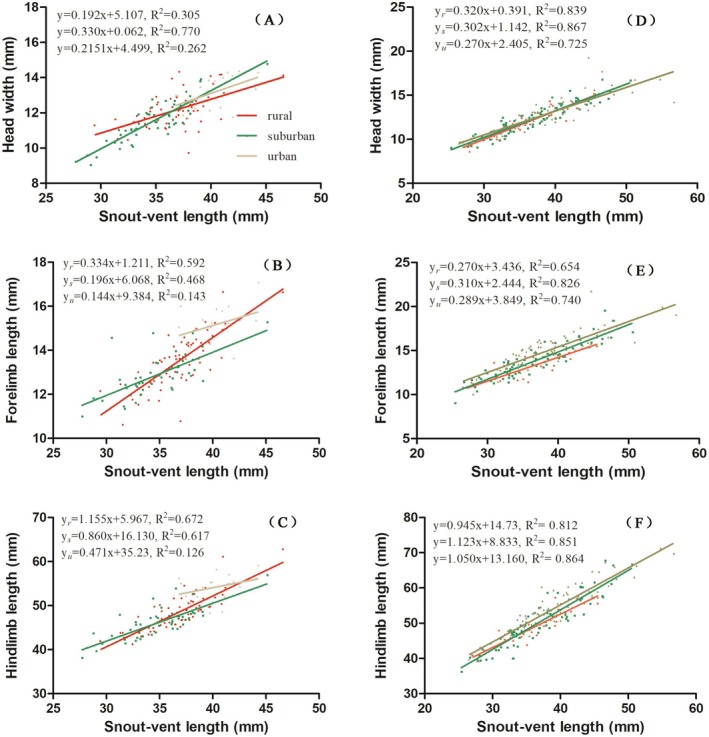
Size scaling rates for head width, forelimb length, and hindlimb length in *F. multistriata* (males: A–C, females: D–F) along the urbanization gradient in Shanghai.

**FIGURE 5:**
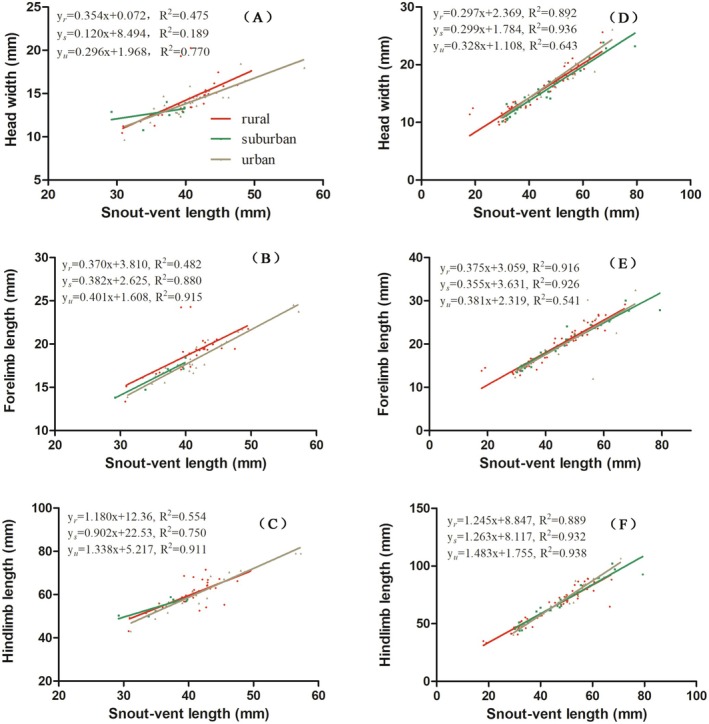
Size scaling rates for head width, forelimb length, and hindlimb length in *P. plancyi* (males: A–C, females: D–F) along the urbanization gradient in Shanghai.

**FIGURE 6:**
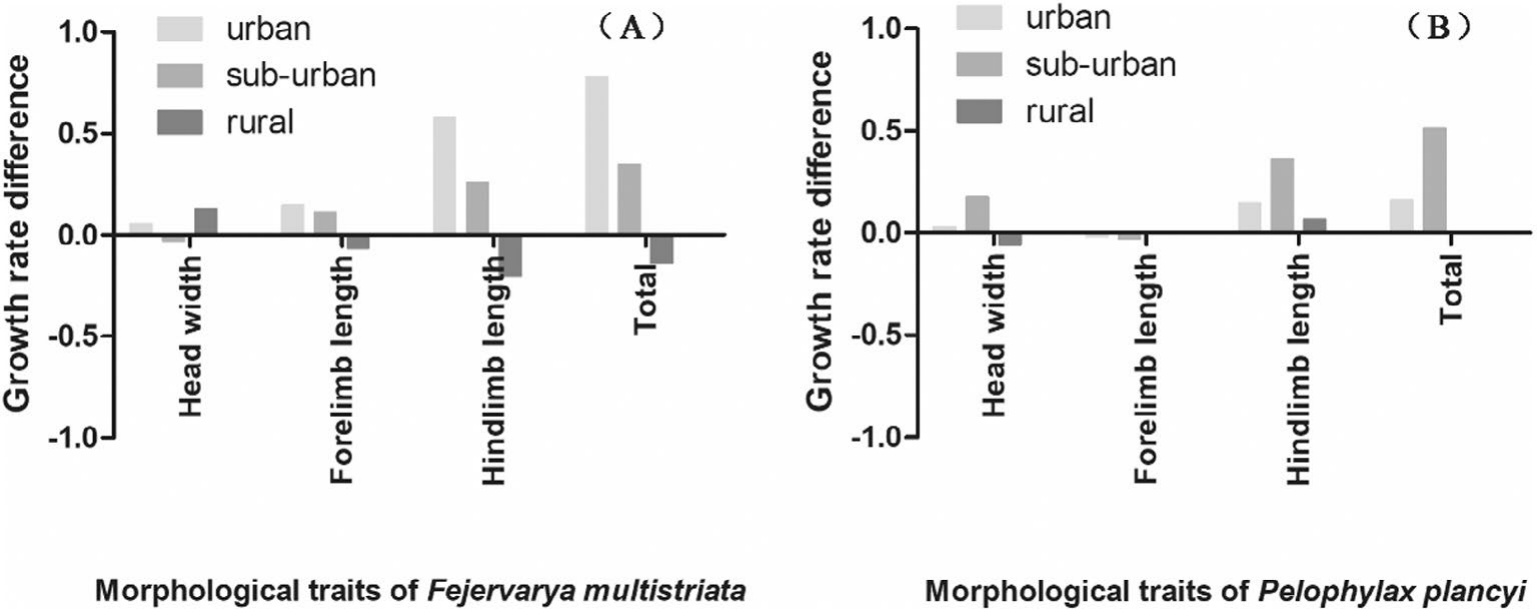
Differences in size scaling rates between females and males (female minus male) for head width, forelimb length, hindlimb length, and total value in *F. multistriata* (A) and *P. plancyi* (B) along the urbanization gradient.

Linear regression analyses of SVL and other morphological traits were conducted for urban, suburban, and rural populations of both sexes in the two anuran species (Figure 4: *F. multistriata* ; Figure 5: *P. plancyi* ). The results showed that the size scaling rates were lower for the forelimb length (0.144) and hindlimb length (0.471) in urban *F. multistriata* males, as well as head width (0.270) in urban females, than those in suburban and rural populations (Table S4). In addition, the differences in size scaling rates between urban females and males were more pronounced for the forelimb length, hindlimb length, and total score for all of the other three morphological traits compared with suburban and rural populations (Figure 6 and Table S5).

In contrast, urban *P. plancyi* males exhibited the highest size scaling rates for forelimb length (0.401) and hindlimb length (1.338), and urban females had the highest size scaling rates for head width (0.328), forelimb length (0.381), and hindlimb length (1.483) (Table S4). Similarly, urban and suburban populations of *P. plancyi* also exhibited higher sexual differences in terms of size scaling rates for other morphological traits compared with rural populations (Figure 6 and Table S5).

## 4 Discussion

### 4.1 Body Size and SSD

Body size shifts in aquatic and terrestrial urban communities have been a popular topic in urban ecology and evolutionary ecology, because they may be affected by metabolic cost changes due to the urban heat island effect and habitat fragmentation (Merckx et al. 2018). In the present study, we showed that the body sizes in both sexes were larger in urban populations of *F. multistriata* and *P. plancyi* compared with rural and suburban populations (Table 1), and similar results were obtained in some previous studies (Cogălniceanu et al. 2021; Favio et al. 2024; Liu et al. 2025). Under limited resources (especially food availability), small population sizes, high intraspecific competition, high mortality, and the urban heat island effect (Thompson et al. 2022; Liu et al. 2025), growth accelerates in both sexes so they could achieve early sexual maturity and gain reproductive advantages in urban environments. Due to the potential plasticity of size in anurans (Laurila et al. 2002; Buskirk 2009), a similar “body size gigantism” phenomenon in anurans based on an island rule has also been observed in fragmented urban habitats (such as urban parks and green areas), which was driven by selective pressures such as immigration filters, resource limitation, and intra‐ and interspecific interactions (Lomolino 2005). Importantly, the morphological variations in anurans due to environmental pressures are crucial for shaping their SSD (Komine et al. 2022; Lorrain‐Soligon et al. 2023).

According to previous studies of SSD in *F. multistriata* and *P. plancyi* (Shou et al. 2005; Shi et al. 2011), females are larger than males in both frog species due to fecundity selection theory, and this pattern was also found in the present study (although significant differences in SVL between sexes were only detected in urban populations; Tables 2 and 3). Furthermore, we observed male‐biased SSD in some morphological traits along the urbanization gradient. For instance, urban *F. multistriata* populations had longer forelimbs in males (Table 2), reflecting an enhanced capacity to retain their grip on a female during amplexus and resist attempted take‐overs by competing males, which may confer reproductive advantages and could have evolved through sexual selection in anurans (Emerson 1991; Mao et al. 2014; Dursun et al. 2022). Similarly, *P. plancyi* males had wider heads in suburban populations, and male individuals had longer hindlimbs than females in suburban and rural populations (Table 3), indicating the potentially stronger foraging and locomotor performance of males to enhance resource acquisition in non‐urban areas (Herrel et al. 2012; Guillot et al. 2016), with possible effects on functions such as feeding or mating (Dursun and Özdemir 2022).

### 4.2 Variations in SSD

Intraspecific differences in SSD along environmental gradients (e.g., geographic or thermal) have been documented in some anuran species (Cox and Calsbeek 2010; Rojas and Endler 2013; Feng et al. 2015; Dursun et al. 2022), but few studies have examined changes in SSD in anurans along an urbanization gradient (Ofori et al. 2025). We found that SSD in terms of SVL was significantly higher in urban *F. multistriata* populations than suburban and rural populations (Figure 2), where the results exceeded previous records (i.e., Shou et al. 2005; SDI = 0.08). By contrast, it was shown that another anuran species, the African common toad ( *Sclerophrys regularis* ), exhibited stronger SSD in suburban populations than urban populations to maximize their fitness due to higher selective pressures in this area (Ofori et al. 2025). Similarly, in reptiles, Bury and ZajĄc (2020) reported that SSD was lower in urban populations of European grass snakes ( *Natrix natrix* ) compared with suburban and rural populations in Europe, and Huang et al. (2025) found that SSD was lower in Dekay's brown snake ( *Storeria dekayi* ) in more urbanized areas of North America, but these findings are inconsistent with our results.

Rensch's rule (Rensch 1950) posits that SSD increases with body size in species where males are larger and decreases where females are larger. Evidence to support this rule was primarily obtained from interspecific studies of male‐biased SSD species (Abouheif and Fairbairn 1997; Székely et al. 2004), although this was also found in female‐biased species (Fairbairn 1997; Székely et al. 2004). The inverse Rensch's rule is common in anurans due to the balance between fecundity selection on females and sexual selection on males (Liao et al. 2013). Intraspecific studies of species with larger females are scarce, but some found patterns that contradicted Rensch's rule (Liao 2013; Liao et al. 2015; Feng et al. 2015). Both of our study species exhibited increased morphological size as the urbanization score increased (Table 1; Zhong et al. 2023; Liu et al. 2025) and female‐biased SSD (Tables 2 and 3), and our results also showed that the intensity of SSD increased with female body size along the rural–urban gradient in both anuran species (Figures 2 and 3), suggesting the inverse of Rensch's rule along the urbanization gradient in Shanghai based on RMA regression (Table 4). These results did not support our first hypothesis that SSD would decrease with increasing urbanization score. Indeed, fecundity selection has been suggested to explain these observations in animals with the inverse of Rensch's rule (Herczeg et al. 2010; Liao et al. 2015), and stronger sexual selection in females may also explain this trend (Dale et al. 2007). Sexual selection of females can emerge from competition for access to mates, breeding territories, and other critical resources for reproduction and food (Clutton‐Brock 2009; Rosvall 2011). Furthermore, sexual selection, fecundity selection, and niche divergence may act alone or in combination on SSD in animals (Fairbairn 1997), but the driving mechanism in anurans along the urban–rural gradient needs to be tested further.

SSD in terms of SVL was stronger in urban *F. multistriata* populations than suburban and rural populations, but no significant difference was found in SSD in *P. plancyi* along the urbanization gradient. These results were consistent with the second hypothesis that SSD would be affected less by urbanization in *P. plancyi* than *F. multistriata* . Indeed, urbanization could potentially influence SSD in anurans. For example, a meta‐analysis of 3500 amphibian species by Pincheira‐Donoso et al. (2021) identified temperature changes due to global warming and latitude as drivers of variations in SSD. Land‐use changes caused by urbanization alter surface and water temperatures (Phelan et al. 2015), such as the urban heat island effect, to potentially impact the intensity of SSD under habitat and resource restrictions. We did not investigate the age of each population in this study, but age structure differences in anurans may also contribute to variations in SSD (Monnet and Cherry 2002; Liao et al. 2013; Reinhard et al. 2015), and body size differences at maturity were identified as most important in some studies (Marzona et al. 2004; Seglie et al. 2010). Considering the variations in the age structure of these two frog species in a previous study (Liu et al. 2025), the relationship between age and SSD needs to be investigated further.

### 4.3 Variations in Size Scaling Rate

Growth can be influenced by changes in food availability and quality, foraging behavior, population density, predator density, and metabolism rate, or differences in habitat and climate, which may then affect the activity levels of anurans (Miaud et al. 2001). However, these conditions can affect both sexes in anurans in a complex manner (especially larval and sub‐adult stages) (Hase et al. 2013), and some researchers found that they could not explain SSD (Reinhard et al. 2015). In the present study, urban populations of *F. multistriata* exhibited a higher size scaling rate for certain morphology traits (e.g., male forelimbs and female head width) (Figure 4), and the growth rate of the hindlimb length in urban males even exceeded that of their body length (Figure 4C), suggesting that urbanization may have driven rapid limb growth. In *P. plancyi* , only the growth rate of the head width was higher in urban males compared with those in suburban and rural populations (Figure 5A), which may also have explained the smaller variations in body size with urbanization in Shanghai.

According to the sexual differences in growth rate found along the gradient, urban populations of *F. multistriata* exhibited greater disparities between sexes than suburban and rural populations (Figure 6A), whereas the differences were minimal in *P. plancyi* (Figure 6B). Similarly, previous studies showed that urbanization had stronger effects on body size, growth rate, and SSD in *F. multistriata* than *P. plancyi* (Zhong et al. 2023; Liu et al. 2025). The greater sexual differences in growth rates may have explained the higher SSD in *F. multistriata* in urban environments. We predict that a particular theory, such as sexual selection theory, fecundity selection theory, or niche divergence theory, may explain the comprehensive impacts on intraspecific variations in SSD. Importantly, we acknowledge this result was descriptive lacking a statistical test due to insufficient sample size in each sex in each urban population.

## 5 Conclusion

We found that the SVL measurements were higher in *F. multistriata* males and females in urban areas compared with suburban and rural areas. In addition, the SVL measurements in *P. plancyi* males were only higher in urban areas compared with those in rural areas. Furthermore, we found that the SSD in terms of SVL was significantly higher in urban populations of *F. multistriata* than suburban and rural populations. All of the SSD results in terms of the SVL, head width, forelimb length, and hindlimb length in these two frog species supported the reverse Rensch's rule based on RMA regression. Moreover, the size scaling rates were higher in the urban and suburban populations of these two frog species than rural populations. However, the low population‐level sample sizes for *F. multistriata* may have limited the precision of the data, and thus more need to be obtained for urban anuran populations. Future research should investigate the mechanisms that determine how multiple factors can drive changes in SSD along urbanization gradients, including sexual age structure differences, male sexual features, female fecundity features, microhabitat, climate, and diet, in order to better understand the evolutionary mechanisms that allow urbanization to affect SSD in animals (Pincheira‐Donoso et al. 2021). In addition, the sample numbers were low for both anurans in urban environments in the present study, so we suggest including more individual anurans in future research.

## Author Contributions

**Ben Li:** conceptualization (equal), formal analysis (equal), funding acquisition (equal), investigation (equal), methodology (equal), resources (equal), writing – original draft (equal), writing – review and editing (equal). **Ningning Liu:** formal analysis (equal), investigation (equal), writing – review and editing (equal). **Shurong Zhong:** formal analysis (equal), investigation (equal), writing – review and editing (equal). **Tianhou Wang:** writing – review and editing (equal). **Rongquan Zheng:** funding acquisition (equal), project administration (equal), writing – review and editing (equal). **Qing Song:** writing – review and editing (equal). **Zhenghuan Wang:** writing – review and editing (equal). **Wei Zhang:** investigation (equal), writing – review and editing (equal).

## Funding

This work was supported financially by the Natural Science Foundation of China (Nos. 32471595, 32370454, 31901099).

## Conflicts of Interest

The authors declare no conflicts of interest.

## Supporting information


**Table S1:** Relationships between snout–vent length with other morphological traits.
**Table S2:** Relationships between snout‐vent length with head width, forelimb length, and hindlimb length in *F. multistriata* and *P. plancyi* based on linear.
**Table S3:** Intensity of sexual dimorphism in *F. multistriata* and *P. plancyi* in terms of snout–vent length, head width, forelimb length, and hindlimb length measurements among urban, suburban, and rural populations (mean and standard deviation).
**Table S4:** Size scaling rates for *F. multistriata* and *P. plancyi* males and females along the urbanization gradient (urban/suburban/rural).
**Table S5:** Differences in size scaling rates between females and males (female minus male) in terms of the head width, forelimb length, and hindlimb length, and total value in *F. multistriata* and *P. plancyi* along the urbanization gradient.
**Figure S1:** Morphological characteristics of *F. multistriata* (left figure) and *P. plancyi* (right figure) in this study.

## Data Availability

All the required data have been uploaded as Tables S1 and S5 and Figure S1, and data in the article.
