# Supplementary material for: Variations in Sexual Size Dimorphism in Two Anurans Along an Urbanization Gradient in Shanghai: Assessment of Rensch's Rule
Source: Ecol Evol. 2026 Jun 15;16(6):e73825. doi: 10.1002/ece3.73825 (PMC13269832; doi:10.1002/ece3.73825)
Supplement: Supplementary file 1 — Table S1: Relationships between snout–vent length with other morphological traits. Table S2: Relationships between snout‐vent length with head width, forelimb length, and hindlimb length in F. multistriata and P. plancyi based on linear. Table S3: Intensity of sexual dimorphism in F. multistriata and P. plancyi in terms of snout–vent length, head width, forelimb length, and hindlimb length measurements among urban, suburban, and rural populations (mean and standard deviation). Table S4: Size scaling rates for F. multistriata and P. plancyi males and females along the urbanization gradient (urban/suburban/rural). Table S5: Differences in size scaling rates between females and males (female minus male) in terms of the head width, forelimb length, and hindlimb length, and total value in F. multistriata and P. plancyi along the urbanization gradient. Figure S1: Morphological characteristics of F. multistriata (left figure) and P. plancyi (right figure) in this study. [file ECE3-16-e73825-s001.docx]

# Supplementary information

**TABLE S1.** Relationships between snout–vent length with other morphological traits in *F. multistriata* and *P. plancyi* (^**^*P* < 0.01).

| Species | Sex | Head width | Forelimb length | Hindlimb length |
| --- | --- | --- | --- | --- |
| *F. multistriata* | Male | 0.734^**^ | 0.800^**^ | 0.831^**^ |
|  | Female | 0.923^**^ | 0.875^**^ | 0.913^**^ |
| *P. plancyi* | Male | 0.838^**^ | 0.875^**^ | 0.811^**^ |
|  | Female | 0.966^**^ | 0.928^**^ | 0.961^**^ |

**TABLE S2.** Relationships between snout-vent length with head width, forelimb length, and hindlimb length in *F. multistriata* and *P. plancyi* based on linear regression.

| Species | Head width | Forelimb length | Hindlimb length |
| --- | --- | --- | --- |
| *F. multistriata* | *y*=0.286*x*+1.676 | *y*=0.310*x* +2.437 | *y*=1.120*x* +8.587 |
| *P. plancyi* | *y*=0.305*x* +1.915 | *y*=0.366*x* +3.399 | *y*=1.279*x* +7.785 |

**TABLE S3.** Intensity of sexual dimorphism in *F. multistriata* and *P. plancyi* in terms of snout–vent length, head width, forelimb length, and hindlimb length measurements among urban, suburban, and rural populations (mean and standard deviation).

| Species | Morphological characteristics | Urban | | Sub-urban | | Rural | |
| --- | --- | --- | --- | --- | --- | --- | --- |
|  |  | mean | SD | mean | SD | mean | SD |
| *F. multistriata* | Snout–vent length | 0.147 | 0.049 | 0.063 | 0.01 | 0.06 | 0.015 |
|  | Head width | 0.006 | 0.061 | 0.034 | 0.03 | 0.100 | 0.056 |
|  | Forelimb length | –0.132 | 0.253 | 0.077 | 0.037 | 0.143 | 0.067 |
|  | Hindlimb length | 0.308 | 0.33 | 0.463 | 0.178 | 0.463 | 0.122 |
| *P. plancyi* | Snout–vent length | 0.287 | 0.046 | 0.127 | 0.106 | 0.201 | 0.069 |
|  | Head width | –0.021 | 0.118 | 0.008 | 0.187 | 0.227 | 0.147 |
|  | Forelimb length | 0.326 | 0.215 | 0.406 | 0.290 | 0.443 | 0.138 |
|  | Hindlimb length | –0.032 | 0.035 | 0.017 | 0.051 | -0.040 | 0.034 |

**TABLE S4.** Size scaling rates for *F. multistriata* and *P. plancyi* males and females along the urbanization gradient (urban/suburban/rural).

| Species | Sex | Head width | Forelimb length | Hindlimb length |
| --- | --- | --- | --- | --- |
| *F. multistriata* | Male | 0.215/0.330/0.192 | 0.144/0.196/0.334 | 0.471/0.86/1.150 |
|  | Female | 0.270/0.302/0.320 | 0.290/0.310/0.270 | 1.050/1.123/0.945 |
| *P. plancyi* | Male | 0.296/0.120/0.354 | 0.401/0.382/0.370 | 1.338/0.902/1.180 |
|  | Female | 0.328/0.299/0.297 | 0.381/0.355/0.375 | 1.483/1.263/1.245 |

**TABLE S5.** Differences in size scaling rates between females and males (female minus male) in terms of the head width, forelimb length, and hindlimb length, and total value in *F. multistriata* and *P. plancyi* along the urbanization gradient (urban/suburban/rural).

| Species | Head width | Forelimb length | Hindlimb length | Total |
| --- | --- | --- | --- | --- |
| *F. multistriata* | 0.055/-0.028/0.129 | 0.146/0.114/-0.064 | 0.579/0.263/-0.205 | 0.780/0.349/-0.140 |
| *P. plancyi* | 0.032/0.179/-0.057 | -0.020/-0.028/-0.005 | 0.145/0.361/0.065 | 0.157/0.512/0.003 |


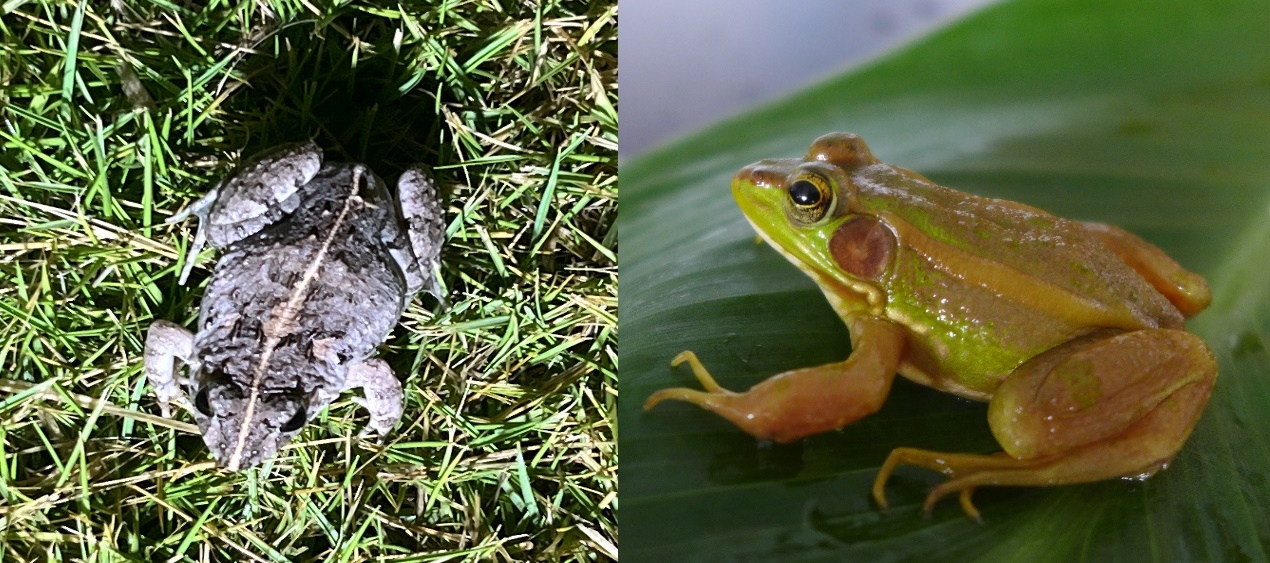
**Figure S1.** Morphological characteristics of *F. multistriata* (left figure) and *P. plancyi* (right figure) in this study.
